# Disseminated Cutaneous Leishmaniasis, a Patient with 178 Lesions Cured with Fluconazole

**DOI:** 10.4269/ajtmh.15-0211

**Published:** 2016-01-06

**Authors:** Anastácio Queiroz Sousa, Margarida M. Lima Pompeu, Richard D. Pearson

**Affiliations:** Federal University of Ceará School of Medicine, Fortaleza, Ceará, Brazil; Hospital São José for Infectious Diseases, Fortaleza, Ceará, Brazil; University of Virginia School of Medicine, Charlottesville, Virginia

**Figure 1.**
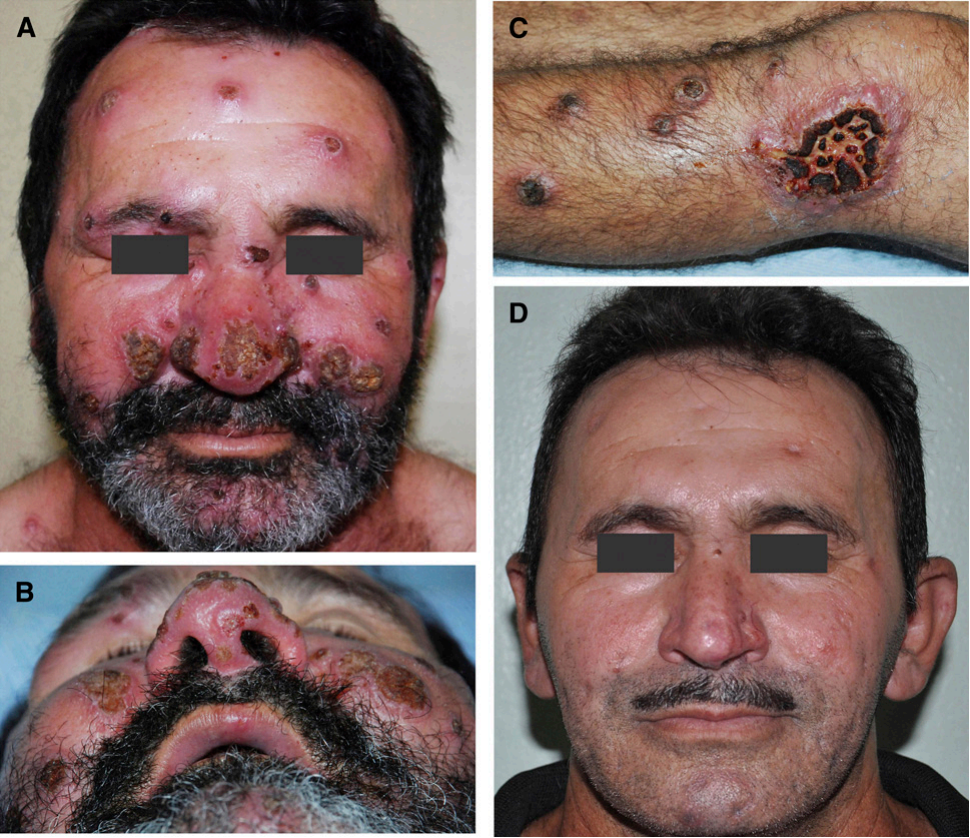
(**A** and **B**) More than 20 crusted and ulcerated lesions can be seen on the face, with involvement of the forehead, eyelid, nose, cheeks as well as the upper lip. (**C**) The larger ulcerated lesion is clearly draining purulent secretion. (**D**) Patient's face after treatment shows that most lesions left no scar.

A 52-year-old male farm worker from Ceará state, northeast Brazil, presented with a 4-month history of skin lesions. Lesions appeared first on the face and legs, than spread across much of his body, including the genitalia, soles of feet, and scalp. He reported daily fevers for the first 2 weeks of illness. On examination the patient had 178 lesions of different sizes and aspects^1^–^3^; ulcerated, crusted (Figure 1A and B

), papular, nodular, and pustular. Many had apparent bacterial superinfections (Figure 1C). The remainder of the physical examination was normal. A complete blood count and liver and kidney function tests were within normal limits. Serological tests for syphilis and human immunodeficiency virus were negative. A skin biopsy imprint revealed amastigotes. Skin culture in Novy-MacNeal-Nicolle medium grew promastigotes characterized as *Leishmania* (*Viannia*) *braziliensis* by isoenzymes. The leishmanin skin test was positive. Bacterial superinfection of the ulcers was treated with cephalexin. Because of a contraindication to treatment with pentavalent antimony (individual > 50 years of age), and lack of medical infrastructure where he lived for providing amphotericin B, the patient was treated with oral fluconazole, 9 mg/kg per day.^4^ Over the following 6-month period of treatment, the lesions healed completely, many leaving no scars (Figure 1D). Disseminated cutaneous leishmaniasis is an uncommon presentation of *Leishmania* (*V.*) *braziliensis* infection. Most of the lesions are thought to be due to bloodstream dissemination, not to multiple sand fly bites. The reason that some people develop disseminated cutaneous leishmaniasis is not fully understood.^3^
